# A Novel Hypothesis: A Role for Follicle Stimulating Hormone in Abdominal Aortic Aneurysm Development in Postmenopausal Women

**DOI:** 10.3389/fendo.2021.726107

**Published:** 2021-10-13

**Authors:** Victoria N. Tedjawirja, Max Nieuwdorp, Kak Khee Yeung, Ron Balm, Vivian de Waard

**Affiliations:** ^1^ Department of Surgery, Amsterdam University Medical Center (UMC), University of Amsterdam, Amsterdam Cardiovascular Sciences, Amsterdam, Netherlands; ^2^ Departments of Internal and Vascular Medicine, Amsterdam UMC, University of Amsterdam, Amsterdam, Netherlands; ^3^ Department of Medical Biochemistry, Amsterdam UMC, University of Amsterdam, Amsterdam Cardiovascular Sciences, Amsterdam, Netherlands

**Keywords:** follicle stimulating hormone, abdominal aortic aneurysm, menopause, women, osteoporosis, macrophages, atherosclerosis, adiposity

## Abstract

An abdominal aortic aneurysm (AAA) is a dilatation of the abdominal aorta, which can potentially be fatal due to exsanguination following rupture. Although AAA is less prevalent in women, women with AAA have a more severe AAA progression compared to men as reflected by enhanced aneurysm growth rates and a higher rupture risk. Women are diagnosed with AAA at an older age than men, and in line with increased osteoporosis and cardiovascular events, the delayed AAA onset has been attributed to the reduction of the protective effect of oestrogens during the menopausal transition. However, new insights have shown that a high follicle stimulating hormone (FSH) level during menopause may also play a key role in those diseases. In this report we hypothesize that FSH may aggravate AAA development and progression in postmenopausal women *via* a direct and/or indirect role, promoting aorta pathology. Since FSH receptors (FSHR) are reported on many other cell types than granulosa cells in the ovaries, it is feasible that FSH stimulation of FSHR-bearing cells such as aortic endothelial cells or inflammatory cells, could promote AAA formation directly. Indirectly, AAA progression may be influenced by an FSH-mediated increase in osteoporosis, which is associated with aortic calcification. Also, an FSH-mediated decrease in cholesterol uptake by the liver and an increase in cholesterol biosynthesis will increase the cholesterol level in the circulation, and subsequently promote aortic atherosclerosis and inflammation. Lastly, FSH-induced adipogenesis may lead to obesity-mediated dysfunction of the microvasculature of the aorta and/or modulation of the periaortic adipose tissue. Thus the long term increased plasma FSH levels during the menopausal transition may contribute to enhanced AAA disease in menopausal women and could be a potential novel target for treatment to lower AAA-related events in women.

## Introduction

An abdominal aortic aneurysm (AAA) is a dilatation of the abdominal aorta with a diameter of ≥ 3 cm, which, after further aortic dilatation, poses a risk for aortic rupture and subsequent death (1). Although the prevalence of AAA is higher in men than in women, the condition in women is more deleterious as they are at higher risk of rupture than men at the same aortic diameter and have increased AAA growth rates than men (2–7). While the higher rupture risk at equal aortic diameters may be explained by the fact that women are in general smaller than men, which suggests a proportionally greater enlargement, this does not explain the enhanced AAA growth rates in women. Therefore, the exact cause of this morbidity in women is not yet fully understood. The hormonal changes during the menopausal transition have been a subject of interest as a possible explanation since women present themselves with AAA at an older age than men (8). However, the role of sex hormones in the progression of AAA in women is inconclusive as described in a review (9) and will be elaborated on further below after briefly addressing current management and characteristics of the pathogenesis of AAA. As hormone replacement therapy in postmenopausal women was not clearly negatively associated with AAA (10, 11), this led to the exploration of an additional (hormonal) factor that may be at play. The current report hence shows the development of AAA in postmenopausal women from another hormonal perspective, leading to our hypothesis that follicle stimulating hormone (FSH) may enhance AAA onset or progression, *via* different mechanisms as outlined below.

## Contemporary Surgical and Pharmacologic Treatment Options for AAA

Elective surgical AAA repair to prevent rupture is currently indicated at an aortic diameter of more than 5.0 cm in women and more than 5.5 cm in men (12). In the acute setting, patients with ruptured AAA also require surgery. The observation that women have higher mortality rates than men after both elective and ruptured AAA repair is worrisome (13, 14). Female sex has been associated with mortality and it has been suggested that it may be a proxy of risk factors that are specific to women (15).

Prior research of *ex vivo* cultures of human AAA tissue, animal AAA models and clinical trials in patients investigated various pharmacological agents as an intervention, which were aimed at inhibiting AAA progression. Outcomes for effectiveness of these drugs include the growth of AAA diameter in mm, AAA characteristics on histology level, or the measurement of AAA-related markers, such as the proteases matrix metalloproteinase 2 (MMP-2) and/or 9 (MMP-9) (16–18). Suggested possible future roles of those pharmacological agents were to be used as prevention in patients at risk for AAA, patients with a small AAA diameter or after surgical repair as additional support (19). Despite extensive AAA research, currently no established drugs are available that can diminish or stabilize AAA growth (19–22), besides cardiovascular risk management that may improve long-term survival of AAA patients (23). Interestingly, diabetes has been reported to be inversely associated with AAA (24, 25). An explanation for this observation from a pharmacological point of view is that diabetes medication, such as metformin, may have played a role through its vascular anti-inflammatory effects and have an effect on other mechanisms that are involved in AAA formation (26–29). As such, in part based on a large retrospective AAA study with 13,834 AAA patients (30), there are currently three trials running using metformin (NCT04500756, NCT04224051, NCT03507413). However, in the large retrospective AAA study, there were only 0.6% women with an AAA and a sub-analysis of the association of metformin prescription with the protection of AAA in women was not shown (30). Since it is not yet clear if metformin is also associated with lower risk of AAA in women, further research to find a potential target for pharmacological treatment is needed, with perhaps a different target in women.

## AAA Histopathology and Pathogenesis

The understanding of the mechanisms involved in AAA pathogenesis has been expanding. Many complex processes have been identified to contribute to the remodelling and weakening of the aortic wall, leading to AAA (1, 22). On a histopathological level, AAA is characterized by smooth muscle cell (SMC) apoptosis, accumulation of inflammatory cells, extracellular matrix (ECM) degradation and oxidative stress (1, 31). However, it seems that not only the abdominal aorta is affected in patients with AAA but also the rest of the cardiovascular system. A study using individuals with asymptomatic AAA from the UK Small Aneurysm Trial showed an association between AAA diameter and cardiovascular and all-cause mortality before aneurysm surgery or rupture and after surgical repair, suggesting that AAA diameter is a marker for progressive cardiovascular disease (CVD) (32). Another study showed that patients with small AAA had a high prevalence of CVD and that patients with small AAA were at increased risk for CVD death (33). Indeed, atherosclerosis in itself is a major contributor to CVD. Although AAA share some common risk factors with atherosclerosis, including advanced age, smoking and hyperlipidaemia (34–36), there was some uncertainty if atherosclerosis has a causal role in AAA or if both conditions coexist (36–38). The current view is that the mechanisms for both pathologies are different, yet that certain risk factors may overlap (36–38). Furthermore, while atherosclerosis does not always precede AAA, it could contribute to AAA through the effects of chronic inflammation (36, 39). A study comparing AAA tissue with atherosclerotic wall samples from the same region showed that AAA distinguishes from atherosclerotic disease by enhanced expression and activation of inflammatory transcription factors (40). This finding is corroborated in a recent study that compared AAA patients with age and sex-matched atherosclerotic patients, and demonstrated that *inflammation* and *calcification* of the aorta was greater in patients with AAA (41). On a histological level, the evidently highly inflamed aorta in AAA shows massive infiltration of inflammatory cells in the outer SMC layers (media) and external collagen layer (adventitia). These inflammatory cells can form lymphoid follicles and some are united into lymph node-like structures (42). During this process, microvessels in the medial and adventitial layer, also known as the vasa vasorum, are surrounded by T-cells, dendritic cells, macrophages and to a lesser extent B-cells (42), revealing that the inflammatory cells in AAA are likely recruited from the vasa vasorum.

## Hormones in Relation With AAA

### Sex Steroid Hormones: Oestrogen and Testosterone

There is an increasing number of studies on hormonal differences in AAA as a potential explanation for the worse morbidity in women compared to men. Many studies have investigated the effects of both endogenous and exogenous oestrogen. The cellular mechanisms of oestrogen in modulating anti-inflammatory and vasoprotective processes in response to vascular injury have been identified in multiple laboratory studies (43, 44). As plasma levels of oestrogen diminish around the menopause (45), AAA has been proposed to be attributed to the decline of the protective effect of oestrogen. In support of this theory, an observational study found that women with earlier menopause were characterized by larger AAA diameters (46). The oestrogen effect has also been demonstrated in various animal AAA models (47–50). Two experimental AAA studies that compared ovariectomized mice with a sham operation group showed specifically an increased expression of MMP-9 in AAA tissue in the ovariectomized group (47, 48). Whereas one study also showed an increase in MMP-2 and the other study found no difference (47, 48). Both are proteases that have been identified to promote the development of AAA (51, 52). The effect of oestrogen supplementation in older female rats however seems to be lost (43, 53). This was demonstrated in a study in which older female rats and young female rats underwent balloon injury of the carotid artery and were treated with either vehicle or oestrogen. Upon injury, oestrogen did not reduce, but actually enhanced neointima formation in older female rats while it did reduce neointima formation in young female rats. Furthermore, oestrogen did not have an inhibitory effect on leukocyte infiltration and mRNA expression of inflammatory mediators in injured arteries as is seen in young female rats (53). The timing hypothesis has been proposed to explain this discrepancy in older *versus* younger subjects. This hypothesis consists of the protective effect of oestrogen/oestrogen and progestin on atherosclerosis when given early after the onset of menopause in women, which is lost when initiated long after the menopause (54). This hypothesis may explain why hormone replacement therapy (HRT) in older postmenopausal women is associated with no benefit or even a slightly higher risk of AAA, whereas HRT was reported to have an inverse association with AAA in women who had a mean age of 43.5 years (10, 11, 55, 56). This complex and inconclusive effect of HRT was also seen in the setting of CVD in postmenopausal women (57–59), which have led to the question if there may be an additional factor that could have contributed to the differences observed after the menopause.

While oestrogen receptors are considered to be protective transcription factors in vascular disease (60), a single administration of testosterone in female neonatal mice increased the AAA incidence compared to mice administered vehicle as neonates in the angiotensin-II induced aneurysm/dissection model (61). In addition, the authors reported that the external diameters of the abdominal aortas and aneurysm pathology of female mice administered testosterone as neonates were similar to those observed previously in adult male mice by the same study group (61). Testosterone is also an essential hormone in women and circulating testosterone plasma levels were slightly lower than those of oestrogen in premenopausal women in a cross-sectional study (62). In a prospective longitudinal study, the total testosterone plasma levels were annually measured in women who underwent menopause and the study reported that the total testosterone levels were unchanged across the menopausal transition (63). Although interestingly, Burger et al. (63) reported higher pre-final menstrual period testosterone levels compared to the levels reported in premenopausal women by Skiba et al. (62). Could the timing of drawing blood partly explain this difference? As oestrogen levels decrease during/after the menopause, the ratio of testosterone to oestrogen increases (64, 65). Testosterone activates the androgen receptor, a transcription factor similar to the oestrogen receptors, that is expressed in many different cell types, including in vasculature and immune cells (66, 67). While some researchers found that pharmacological blockade of the androgen receptor or deficiency of the receptor in male mice attenuated AAA formation (68) and castration of male mice with established AAA promoted aneurysm stabilization (69), others find the opposite (70). Also in humans there is controversy as to the beneficial or detrimental roles that testosterone (replacement therapy) plays in vascular disease (60, 71–74). Since testosterone replacement therapy is provided nowadays in women and men for various reasons (72, 75), for example in postmenopausal women for treating hypoactive sexual desire disorder (76), its effect on AAA development may become evident in the future. So far there is only an association of low testosterone levels in men with AAA (77). A more elaborate overview of sex hormones in AAA is given in a review by Makrygiannis et al. (78). Taking these findings together, the inconclusive effect of oestrogen and the as-yet unidentified role of testosterone on AAA in postmenopausal women have led to the exploration of the potential impact of other hormones.

### Gonadotropins: Follicle Stimulating Hormone and Luteinizing Hormone

Upon a reduction in oestrogen production in the menopausal transition, there is a gradual increase in follicle stimulating hormone (FSH) and luteinizing hormone (LH) levels. FSH and LH are hormones produced and released by the anterior pituitary gland. They are gonadotropic hormones because they stimulate the function of ovaries in females and testis in males with the purpose of reproduction. In women, the hormones activate the ovaries to produce oestrogen, androgen and progesterone to stimulate follicular growth, maturation and ovulation (79, 80). In men, both gonadotropins are necessary for spermatogenesis. FSH and LH stimulate the production of inhibin and testosterone by the Sertoli and Leydig cells, respectively (81). During the menopausal transition period in women, when the follicle reservoir in the ovaries is diminishing, plasma FSH and LH levels become elevated to maintain follicle development and compensate the decrease in oestrogen production (82). Once the ovaries are completely depleted of follicles, the negative feedback by oestradiol on the hypothalamus and/or pituitary gland is missing, which leads to excess gonadotropin production (83). Whilst FSH and LH are mostly known for their gonadal functions, FSH and LH receptors (FSHR and LHR) are also found in numerous cell types unrelated to sexual development (84, 85), which suggests there are roles for FSH and LH beyond its gonadal function in women. These FSH and LH receptors may become extensively activated upon chronic high FSH and LH blood levels seen during the menopause. In this respect, women with Turner Syndrome, a genetic condition due to a chromosomal abnormality where (part of) one of the X chromosomes is missing, is partly characterized by hypergonadotropic hypogonadism (86). These patients are at risk to develop aortic dilatations and associated dissections in the ascending and descending thoracic aorta (87, 88), and multiple other comorbidities including hypertension, increased risk of bone fracture, impaired glucose tolerance, increased total fat mass, and a range of congenital heart diseases and autoimmune diseases (86). Would it be possible that FSH and LH are not innocent bystanders, but are active participants in aneurysm formation? Indeed, high plasma LH has been associated with increased ischaemic heart disease and AAA in older men (77, 89). Contributing to CVD, a study with ovariectomized female mice showed that administration of LH promoted atherosclerosis (90). For the interested reader, a number of extragonadal sites of LHR expression is summarized in a review on the extragonadal effects of LH and chorionic gonadotropin (CG) (85), including the (foetal) adrenal gland and kidneys, and other parts of the reproductive tract such as the cervix, oviduct, endometrium and myometrium (91, 92). However, as contemporary studies demonstrated a possible role for FSH in conditions which were once thought to be solely a result of the diminished protective effect of oestrogen in women during the menopausal transition (84), we similarly focused in the current report on the potential role of the extragonadal FSHR in AAA. The previously described potential role of FSH with respect to postmenopausal osteoporosis and cardiovascular disease can be appreciated in an excellent review by Zhu et al. (84). Furthermore, for the interested reader, we refer to a recently published chapter that has been dedicated to various aspects of FSH in fertility that extends to the potential actions beyond fertility (93). We summarized some important studies on the extragonadal FSHR in **Supplemental Table 1** which may be relevant for the current report. Although Chrusciel et al. reviewed some discrepancies found in FSH research, suggested to be partly based on species-specific findings and/or the use of poorly validated antibodies (94), with the current knowledge, there are signs that there is a role for FSH in various diseases in postmenopausal women.

## Hypothesis: FSH Enhances the Onset or Progression of AAA

**Figure 1:**
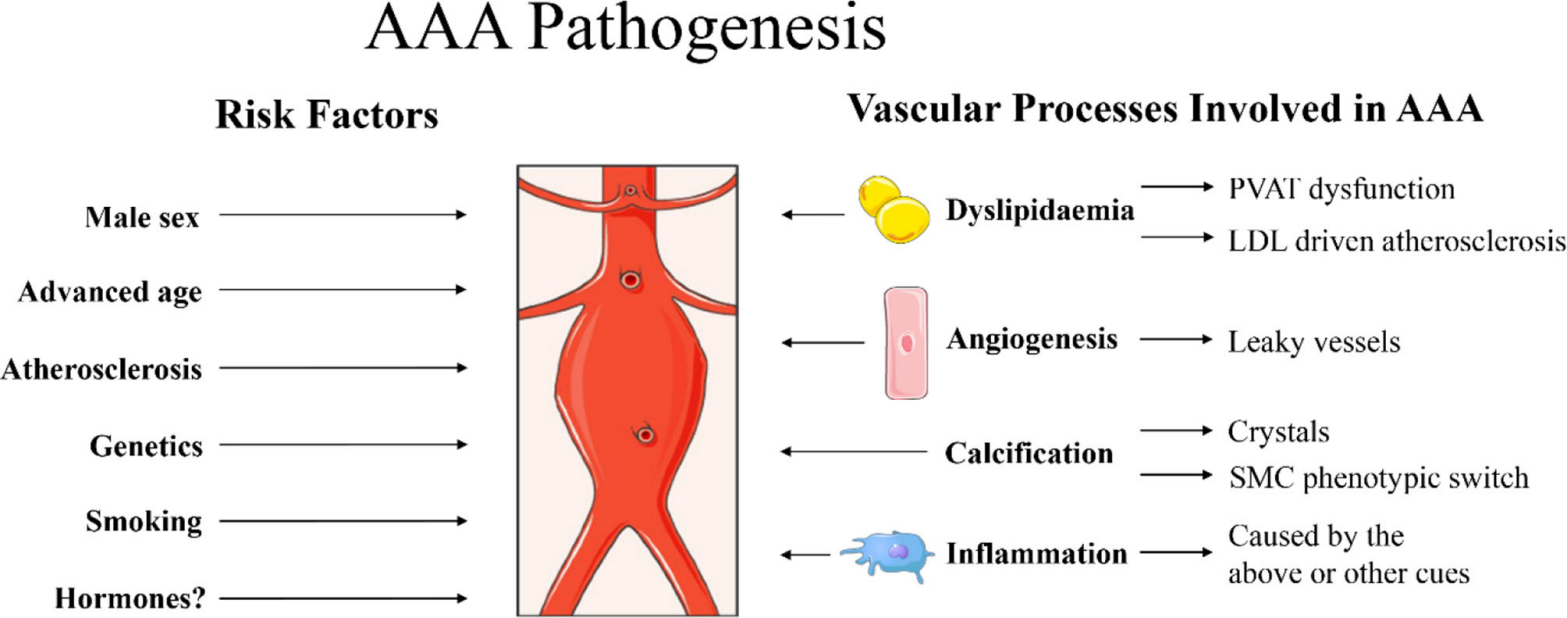
Summary of risk factors for abdominal aortic aneurysm (AAA) pathogenesis as described in the current report. Male sex, advanced age, atherosclerosis, genetics and smoking are identified clinical risk factors for AAA (1). Hormones are also suggested to play a role, although the exact mechanisms are still under investigation. Specific processes at play in the vascular wall that may promote AAA include dyslipidaemia, promoting dysfunctional perivascular adipose tissue (PVAT) (99, 100) and low-density lipoprotein (LDL) driven atherosclerosis (39). Furthermore, angiogenesis [newly formed angiogenic vessels with increased permeability that can result in vascular leakage (101, 102)], calcification (41) [with crystals causing damage to the vascular wall and/or vascular mineralization through smooth muscle cell (SMC) phenotypic switch into osteoblast-like cells (103)] and massive inflammation (1) are also observed in aneurysmal tissue. Images were used from Smart Servier Medical Art.

**Figure 2:**
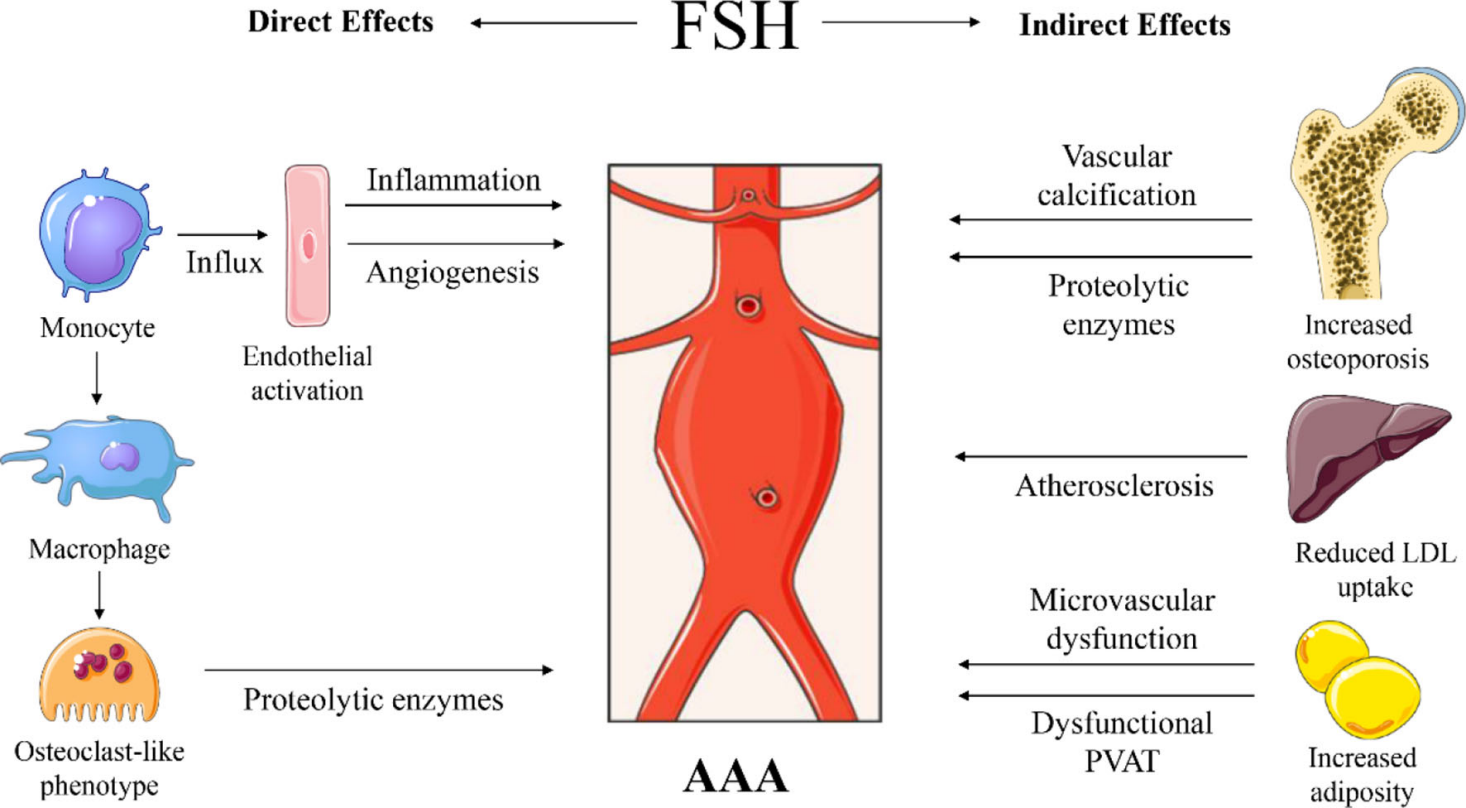
Proposed sites of action and mechanisms of follicle stimulating hormone (FSH) on abdominal aortic aneurysm (AAA) development. Direct effects through increased influx of monocytes in the vascular wall, through FSH-mediated endothelial cell activation and angiogenesis. FSH can stimulate macrophages to become osteoclast-like cells that will produce proteolytic enzymes. Indirect effects through FSH-induced osteoporosis associated with vascular calcification and potential systemic release of proteolytic enzymes. FSH reduces low-density lipoprotein (LDL) cholesterol uptake by the liver and increases cholesterol biosynthesis, causing increased LDL in the circulation, contributing to atherosclerosis. FSH induces adipogenesis, thus adiposity with potential microvascular dysfunction and impaired perivascular adipose tissue (PVAT) function. Images were used from Smart Servier Medical Art.

FSH is a glycoprotein polypeptide hormone and can exert its effects on cells expressing the FSH receptor (FSHR) on their cell membrane. FSH primarily stimulates signalling in the granulosa cells in ovaries and Sertoli cells in testis (84). Some extragonadal sites with FSHR expression have been identified that may contribute to disease, upon chronic stimulation. The most profound examples are postmenopausal CVD and osteoporosis, which were thought to be primarily related to reduced oestrogen levels (95, 96). New insights however reveal that FSH can contribute to the development of both conditions (84). Therefore, we believe that the elevation of circulating FSH for a number of years spanning the menopausal transition may also have consequences for the development of other diseases affecting postmenopausal women. An example of extragonadal FSHR expression is in stem cells in bone marrow in mice (97). Upon stimulation with FSH in women, those stem cells were shown to be mobilized to the circulation (98), which thereby reach all organs. We hereby hypothesize that there may be direct and/or indirect effects of FSH that contribute to AAA development or progression. First, a summary of known mechanisms promoting AAA development is provided in **Figure 1**. The potential effects of FSH on AAA are described in the following paragraphs and summarized graphically in **Figure 2**.

## The Direct Effects of FSH on the Vascular Wall

### Macrophages

Macrophages are inflammatory cells that can play a pro-inflammatory or anti-inflammatory role and as such can regulate tissue injury and repair (104). In AAA, there are many different processes ongoing that macrophages are also thought to be involved in, such as ECM remodelling, inflammation and oxidative stress (105). Dependent on (micro)environmental cues, macrophages can adopt different phenotypes. As such, if disturbed, an imbalance in the identity and function of the macrophage can result in (vascular) disease (106, 107). Interestingly, macrophages are involved in vascular calcification (108). In AAA and atherosclerosis calcification is often observed, and in atherosclerotic plaques the contribution of macrophages to vascular calcification is proposed to be *via* disrupted signalling in macrophages that impairs their osteoclast-like activity, and/or *via* macrophage-derived cytokines that induce osteogenic differentiation and mineralization of SMC (109, 110). During SMC calcification in the aortic root in mice, oxidative stress induced the expression of receptor activator of nuclear factor-κB ligand (RANKL) *via* runt-related transcription factor 2 (Runx2; a transcription factor associated with osteoblast differentiation) in SMC. This upregulation increased bone marrow derived monocyte migration and differentiation into tartrate-resistant acid phosphatase (TRAP) positive osteoclast-like cells (111). This finding suggests a direct role for osteoclast-like cells in vascular calcification. Possibly, there is also a role for osteoclastogenesis in AAA (112, 113). AAA often contain advanced stages of atherosclerosis with calcification. Due to the abundance of macrophages in atherosclerotic areas in AAA, these cells can be a potential local source of osteoclast-like cells (112, 114). The role of osteoclast-like cells in AAA has been demonstrated in an interesting study using human aortic tissue, the murine RAW 264.7 macrophage cell line, and two aneurysm mouse models using calcium chloride (CaCl_2_) and angiotensin II (113). The authors showed that the majority of the macrophages in human AAA tissue was differentiated into osteoclast-like cells (TRAP positive) and that they produced significantly greater MMP-9 activity compared to the undifferentiated macrophages (113). Furthermore, the authors showed that Tumour Necrosis Factor alpha (TNFα) in the presence of calcium phosphate enhanced macrophages to differentiate into osteoclast-like cells (TRAP-positive cells) *in vitro* (113). This suggests that the deposition of calcium phosphate is a prerequisite for the formation of osteoclast-like cells from macrophages *via* TNFα in AAA.

The macrophages in the aortic wall in AAA are mostly derived from peripheral blood monocytes (105), a cell-type on which the FSHR has been identified previously (115). A study on bone loss, using mice, an *in silico* model and *in vitro* data, suggested that stimulation of macrophages with FSH resulted in increased TNFα expression that increased the osteoclast precursor pool (116). FSH could possibly have the same effect in the vascular wall since vascular calcifications in atherosclerotic plaques resemble bone calcifications as the organic matrix and calcium phosphate mineral atomic interface has been suggested to be similar in both conditions (117). Thus if we would extrapolate the findings in the setting of bone loss to the (calcified) vascular wall, FSH stimulation could promote macrophage differentiation into osteoclast-like cells *via* TNFα, and subsequently contributed to ECM degradation in AAA.

Next to changing the macrophage phenotype to an osteoclast-like cell, can FSH also stimulate the newly formed osteoclast-like cells directly to become more proteolytic? Indeed, FSH stimulation of RANKL-induced osteoclast-like cells (derived from RAW264.7 macrophages) induced a dose-dependent increase in mRNA expression of RANK, TRAP, MMP-9 and cathepsin K (118). However, RANKL was not upregulated in end stage aneurysmal tissue (113), suggesting that osteoclastogenesis in AAA may not be RANKL-induced or may have occurred at an earlier disease stage.

These reports may indicate a role for FSH in the transdifferentiation of macrophages into osteoclast-like cells in the calcified aorta through TNFα. Once activated, an excessive production of proteases such as MMP-9 and cathepsin K, similar as in bone, can potentially degrade the aortic ECM, leading to AAA progression. Both MMP-9 and cathepsin K are higher in human AAA tissue compared to the atherosclerotic aorta (119). Evidence of the extrapolation of the effects of FSH on macrophages and osteoclast-like cells from an *in vitro* setting into one *in vivo* in human AAA however requires further investigation.

### Endothelial Cells

In addition to FSHR expression on monocytes and osteoclasts, FSHR has been found on endothelial cells. Endothelial cells form the inner lining of the vasculature and are responsible for a proper barrier function and influx of inflammatory cells, amongst many other functions (120). FSH stimulation was observed to promote vascular endothelial adhesion molecule-1 (VCAM-1) expression on endothelial cells, which is a protein involved in monocyte influx through the endothelial cell layer into the tissue (121). This finding suggests a functional FSHR on endothelial cells and may promote the pro-inflammatory influx of cells into the aortic vessel wall. Furthermore, FSH was shown to induce a proangiogenic response similarly effective as vascular endothelial growth factor in cultured endothelial cells (122). These findings were further substantiated by showing that the FSHR is present on endothelial cells in angiogenic blood vessels in different types of tumours (123). In the aorta, the microvascular bed responsible for ample nutrient and oxygen delivery is the vasa vasorum. Upon vascular wall damage, neovascularisation in the arterial wall in AAA is common and seems to be an ongoing process to promote cellular survival in areas of decreased nutrient and oxygen supply (124, 125). Inflammatory cues are also triggers for neovascularisation as is observed in atherosclerotic plaques (101). Newly formed angiogenic vessels have an increased permeability that results in vascular leakage and enhanced inflammatory cells migration into the tissue (101), that may promote the inflammatory status of the AAA. This could possibly contribute to a vicious cycle of angiogenesis and inflammation in AAA. Angiogenesis in AAA has mainly been observed in the SMC-rich medial layers (126) and a correlation between medial neovascularisation and inflammatory infiltration in AAA has been observed (102). As such, angiogenesis should be considered a sign of disease progression as increased gene expression of angiogenic factors was observed at the edge of ruptured AAA compared to other sites within the ruptured AAA (102). In line with the angiogenic factors at the edge in ruptured AAA, the density of microvessels in the medial layer of AAA was significantly increased and the microvessels had smaller diameters (102). Taken together, if FSH contributes to increased VCAM-1 expression and enhanced angiogenesis, this may lead to increased inflammation in the aortic media and subsequent AAA progression.

## Indirect Effects of FSH on AAA

### Osteoporosis and Vascular Calcification

Osteoporosis is characterized by decreased bone mass density (BMD) and discrepancies in the rate of bone resorption (osteoclasts) and formation (osteoblasts), favouring resorption (127). Although seemingly paradoxical, osteoporosis has been associated with arterial calcification in epidemiological studies (128). Vascular calcification is characterized by the pathological deposition of calcium phosphate crystals (129). The observation that both conditions coexist suggest a common mechanism and there are a number of theories on the development of vascular calcification (129, 130). While vascular calcification was believed to be a passive process, comprising the precipitation of calcium and phosphate (131), more recently, it is considered to be an active process, consisting of a regulated process between the induction of osteogenesis and loss of inhibitors of mineralization (131). Changes in blood and urinary calcium have been observed in disease states where bone turnover is increased such as in hyperparathyroidism (132), but could perhaps to a lesser extent also be applicable in osteoporosis. Elevated calcium and phosphate levels could exacerbate vascular calcification (129, 133–135). The accumulating evidence around the mechanisms of osteoporosis and vascular calcification is too extensive to cover in the current work. Although the exact and highly complex mechanisms are as-yet to be determined, possibly the systemic imbalance of bone minerals and associated regulatory factors in the process of osteoporosis can be associated with vascular calcification.

As such, aortic calcification has been seen in relation with postmenopausal osteoporosis. In postmenopausal women, lower metacarpal bone mass and density was associated with a higher degree of abdominal aortic calcification (136), even when adjusted for age (137). Another study suggested a possible role of hormonal factors that are unique to women, showing that after adjustment for age and other confounders, a significant association between vascular calcification of the abdominal aorta and lower bone mass was seen in women and not in men (138). This potential relationship with a hormonal aspect coincides with an older case-control study, showing that the prevalence of aortic calcification was higher in men than in women <65 years, yet after the age of 65, women had more vascular calcification than men (139). With a change in prevalence between the sexes after the age of 65 years, the hormonal alterations during the menopause are of interest. Although the exact mechanism is still under investigation, perhaps there is a role for FSH linking osteoporosis and aortic calcification in postmenopausal women as a shared mechanism. For postmenopausal osteoporosis treatment, denosumab acts by interrupting RANKL-RANK signalling in osteoclasts (140). A role for FSH in osteoporosis may be through involvement of RANKL-induced osteoclastogenesis as at perimenopausal concentrations (50 mIU/ml) FSH can induce increased RANK expression in peripheral blood mononuclear cells (141). In line hereof, FSH treatment of murine bone marrow cells that were differentiated with RANKL and CSF-1 into osteoclasts, resulted in increased osteoclastic differentiation. Subsequently, when antibodies were used against FSH, the FSH-induced osteoclast formation was inhibited. The authors also compared the formation of TRAP+ osteoclasts after RANKL treatment between FSHR^-^/^-^, FSHR^+^/^-^ and wild type mice. In mice devoid of the FSHR, the formation of osteoclasts was significantly attenuated (142). Given the presence of the FSHR on murine and human osteoclasts, a direct role for FSH may be suggested (115, 142, 143). Thus the question arises how the consequences of enhanced osteoporosis and aortic calcification could be responsible for AAA progression.

Interestingly, an elevated degree of calcification of the aortic wall was seen in symptomatic and ruptured AAA patients compared to asymptomatic AAA patients (144), which suggests that aortic calcification is associated with a severe AAA phenotype. Although another study does not reinforce this theory (145), in a murine AAA model, promoting aortic medial calcification with calcium phosphate enhanced AAA severity (146). The phenotypic switch of SMC into osteoblast-like cells has been identified as an important factor in this matter in relation to AAA (103). An *in vitro* and *in vivo* study showed that under calcifying conditions, aortic SMC lost their SMC markers SM22α and SM α-actin, and gained osteogenic markers (alkaline phosphatase, osteocalcin and osteopontin) (147). Calcification consists of macrocalcification (≥ 50 µm crystals) and microcalcification (< 50 µm crystals), in which the latter has been seen to be more prevalent in mouse and human AAA and is suggested to associate positively with AAA formation (148). More specifically, SMC microcalcification mediated *via* Runx2 seems to precede AAA and its presence can further enhance inflammatory cytokines, MMPs or activation of the NLR family pyrin domain-containing 3 (NLRP3) inflammasome pathway (148). A potential mechanism of AAA development in the calcified aorta is that the calcium crystals can physically damage the aorta, which attracts inflammatory cells. Otherwise, the calcification cues may induce macrophages phenotypic switching into the previously mentioned osteoclast-like cells that can produce various proteolytic enzymes, as an association between calcification and elastic lamina degradation in the aortic ECM has been seen in a murine model (149). A role for osteoclasts was demonstrated in a murine AAA model with calcium phosphate. When bisphosphonate (a drug to treat osteoporosis) was administered to bind to calcium phosphate, this resulted in the inhibition of vascular osteoclastogenesis (113). Could FSH possibly also affect these osteoclasts? Although there are signs that aortic calcification may be an important preceding factor, how this may exactly affect aneurysm development remains to be elucidated.

Alternatively, during bone resorption as in osteoporosis, osteoclast markers cathepsin K and TRAP can be found in the circulation (150). The higher production of these proteins during osteoporosis is reflected in the finding that postmenopausal women with osteoporosis have higher circulating levels of cathepsin K (151, 152). Cathepsin K is a known protease which is involved in AAA (119) and may not only be produced locally by activated (osteoclast-like) macrophages, but could possibly also come from the circulation.

### Lipid Metabolism: Cholesterol Accumulation and Adipogenesis

Another indirect effect of FSH may be *via* the liver. The liver plays a key role in lipid homeostasis, by taking up cholesterol from the circulation, and to be disposed of *via* bile and faeces (153). The FSHR is present on human hepatocytes and it has been reported that FSH attenuated endocytosis of cholesterol-rich low density lipoprotein particles (LDL) by reducing the LDL receptor (LDLR) expression in liver tissue (154). The downregulation of LDLR expression consequently reduces LDL uptake from the circulation. This increases serum LDL levels and subsequently promotes LDL accumulation in the arteries enhancing arterial inflammation (154). Furthermore, FSH regulated cholesterol biosynthesis by inducing 3-hydroxy-3-methylglutaryl coenzyme A reductase (HMGCR) and sterol regulatory element-binding protein 2 (SREBP-2) expression in murine hepatocytes and human HepG2 cells, further enhancing cholesterol blood levels (155). Both processes contribute to atherosclerosis, which may affect AAA development. The atherosclerotic plaques will become more advanced, promoting vascular inflammation and calcification (156, 157).

Adipose tissue has also been reported to be FSH sensitive. White adipocytes function as storage for lipids (triglycerides), while brown adipocytes burn these lipids to generate heat (158). In ovariectomized mice that have increased FSH plasma levels, blocking the FSHR with an FSH antibody resulted in reduced adiposity and increased production of thermogenic adipose tissue (159). The localization of the FSHR on adipocytes implicated a direct role for FSH in adipogenesis (159). If these findings were to be extrapolated to humans, FSH could enhance the accumulation of body fat, which is associated with CVD and metabolic disease in postmenopausal women (160). If FSH contributes to body fat accumulation leading to obesity, then microvascular dysfunction throughout the body may be present as obesity is associated with microvascular dysfunction, including endothelial dysfunction (161–163). Several mechanisms have been identified that can modulate the microvasculature; in part this is affected through visceral adipose tissue derived inflammatory adipokines (161, 164). These changes in the microvasculature can result in impaired tissue oxygenation and low grade inflammation, although the exact mechanism and impact of obesity on the microcirculation can differ between organs (161). As such, possibly (the endothelial cells of) the vasa vasorum oxygenating the aorta can similarly be affected.

Furthermore, changes in lipid accumulation or lipid serum levels can also affect perivascular adipose tissue (PVAT) (161, 162). PVAT is adipose tissue surrounding arteries and is critical to maintain the normal functional status of the vasculature, partially through secretion of paracrine factors (165). However, dysfunctional PVAT including obese or aged PVAT can induce abnormal changes and vascular pathology (165). The alterations in PVAT induced by obesity may thus have consequences. In obese rats with metabolic syndrome, PVAT mass was increased and PVAT-derived cytokine leptin contributed to SMC phenotypic switching in vascular remodelling (99). A potential direct link through PVAT and AAA development was suggested in the angiotensin II-induced AAA mouse model. Under obese conditions, secretion of platelet-derived growth factor-D (PDGF-D) by PVAT contributed to AAA formation in these mice through adventitial fibrosis and inflammation (100). In humans, a relationship between the alteration in PVAT and AAA has also been suggested (166–168). This was reflected in the findings that in AAA patients compared to controls, PVAT had a higher density on CT-scans (differences in PVAT quantity) and an increased pro-inflammatory and MMP gene expression profile (166, 167). Moreover, in human AAA tissue there is an increase in adventitial adipocyte clusters and higher expression of adipogenic transcription factors (169). Interestingly, AAA-derived mesenchymal cells showed enhanced adipogenic potential in culture, compared to control aortic tissue-derived cells (169). Lastly, the significant enrichment of adipogenesis in ruptured AAA supports an association between the extent of PVAT remodelling and rupture (169). Thus, the extragonadal effects of FSH leading to increased cholesterol levels and adiposity in postmenopausal women could have indirectly led to enhanced atherosclerosis with arterial inflammation and calcification, dysfunctional vasa vasorum and PVAT, all potentially affecting AAA development.

## Discussion

The current report highlights the potential extragonadal roles of FSH in AAA progression, which may in part explain the severe course of AAA pathogenesis in postmenopausal women. Improving our understanding of the mechanisms underlying AAA development in women should lead to personalized treatment in women in the future. While oestrogen has been believed to protect premenopausal women from AAA, HRT supplementation in postmenopausal women was not associated with protection against AAA. This could in part be explained by the “timing hypothesis theory”, as discussed earlier. However, there may be an additional hormonal factor that contributes to the more severe AAA phenotype in postmenopausal women, which is in line with newly gained insights of the role of FSH in postmenopausal osteoporosis and CVD. Our hypothesis is that the chronically high FSH plasma levels during the menopausal transition may directly and/or indirectly promote AAA severity. A direct role for FSH in the calcified aorta may be in triggering the macrophages to differentiate into osteoclast-like cells, which through their proteolytic activity can degrade the ECM of the aorta. Secondly, FSH can induce endothelial cells to become angiogenic and to express adhesion molecules that attract inflammatory cells (121, 122). For the indirect role, FSH is a newly identified key player in postmenopausal osteoporosis (84), which is associated with aortic calcification. Since vascular calcification is associated with enhanced AAA development, osteoporosis may have an indirect role in promoting AAA progression through aortic calcification. If and how osteoporosis and vascular calcification are causally related has yet to be determined. Furthermore, FSH can downregulate the expression of the LDL receptor and increase cholesterol synthesis in the liver, thereby enhancing circulating LDL which can accumulate in the aortic wall and increase atherosclerotic burden (154). Although there is no causal relation between atherosclerosis and the development of AAA, both conditions carry the same risk factors (34, 35). As atherosclerosis is a risk factor for AAA, this may aggravate AAA development by promoting aortic inflammation. Lastly, FSH can stimulate adipogenesis (159), which may induce adiposity with subsequent increased risk of microvascular dysfunction, potentially affecting the vasa vasorum. Furthermore, obesity has been associated with dysfunctional PVAT, which can secrete disease-promoting factors that may affect the aneurysmal vascular wall.

The current report focused on the hormonal impact on AAA progression in women. However, there is also evidence that hormonal factors play a role in the development of AAA in men. As the condition primarily affects men, testosterone has been thought to attribute to AAA as demonstrated in murine studies with male mice (68, 69), and even in female mice administered testosterone as neonates (61). However, a recent case-control study comparing 65-year old men with and without AAA found that higher oestrogen, higher progesterone and lower testosterone levels were associated with AAA (170). The association of low testosterone levels with AAA is in line with an earlier clinical and murine study (70, 77). This discrepancy in findings reflects the complexity of characterizing AAA development. As male sex is a known risk factor for AAA, this could be easily explained by hormonal differences between women and men. However, a genetic component may also be involved, demonstrated in an angiotensin II-induced AAA mouse model using phenotypic female mice with either sex chromosome XY or XX (171). The authors showed that sex chromosomes influence the gene expression profiles of abdominal aortas. In XY mice specifically, an increase in the expression of inflammatory pathway genes was observed. Interestingly, female XY mice exposed to testosterone had an increased rupture risk of AAA compared to female XX mice (171), suggesting that testosterone may have a different impact in men than in women.

Furthermore, as lifestyle aspect, smoking is an important risk factor for AAA, which is reported to be more prevalent in men across all age groups (172). However, for women who do smoke, the impact on AAA risk seems to be larger than for men (173). A complex interplay of various factors including hormonal, genetic and lifestyle elements seem to contribute to the differences in the development of AAA in men compared to women. However, there may still be other (as-yet unknown) factors that play a role in the sex-disparity of AAA.

Currently, no studies on the presence of the FSHR in aortic tissue and/or the potential effects of FSH on AAA have been conducted and there are some aspects to take into consideration when anticipating this line of research. While many researchers confirmed the presence of FSHR in extragonadal tissues on both protein and mRNA level, there are also studies that could not detect the FSHR as outlined in **Supplemental Table 1**. As the extragonadal FSHR may have a different splice variant (115), this may have challenged the detection of FSHR mRNA with primers that include exon 9 in one study (174). Alternatively, another study that could not detect the FSHR, used male osteoclasts (175). Could male sex have influenced this issue possibly? Furthermore, differences between experimental designs also affect results, reflecting the complexity of assessing the hormonal impact. For example, while transgenic mice with high FSH levels and normal LH levels showed an increase in bone mass, without detection of FSHR in bone (174), another study using FSHR^-^/^-^ mice with high FSH levels demonstrated normal bone mass, and partial FSH deficiency increased bone mass (143).

As a first step in the investigation of a possible direct effect of FSH on the aorta, the mRNA expression or localization of the FSHR in aortic tissue should be assessed, to reveal if there are differences in FSHR gene expression or density on cells in the aorta and/or number of cells positive for FSHR, between AAA and non-diseased aortic tissue. Furthermore, the various cell types present in the aorta could be stimulated *in vitro* with/without FSH to determine the effect of FSH on those cells by measuring the differences in gene expression of e.g. inflammatory/osteoblast/bone resorption markers, depending on the cell type. The indirect role of FSH on AAA may be more difficult to capture. For example, in a mouse model of AAA with female mice, it would be interesting to explore if FSH-induced osteoporosis resulted in larger AAA diameters with enhanced AAA histopathology characteristics and microcalcification compared to female mice without osteoporosis. In humans, one may investigate if postmenopausal women with osteoporosis have larger AAA diameters than postmenopausal women without osteoporosis, indicating a severe AAA phenotype. Some challenges in detecting the FSHR and its effects on extragonadal cells have been elaborated on thoroughly in a previous review by Chrusciel et al. (94). Nevertheless, the role for FSH in AAA pathogenesis seems worth exploring by further fundamental and clinical research following the above pathways, as an attractive potential target to reduce the AAA-related morbidity in women.

## Data Availability Statement

The original contributions presented in the study are included in the article/**Supplementary Material**. Further inquiries can be directed to the corresponding author.

## Author Contributions

VT, MN, RB, and VW contributed to the concept of the work. VT wrote the manuscript with support from VW who supervised the writing process. All authors contributed to the article and approved the submitted version.

## Funding

VT is funded by the AMC Foundation. MN is supported by a ZONMW VICI grant (number 09150182010020).

## Conflict of Interest

The authors declare that the research was conducted in the absence of any commercial or financial relationships that could be construed as a potential conflict of interest.

## Publisher’s Note

All claims expressed in this article are solely those of the authors and do not necessarily represent those of their affiliated organizations, or those of the publisher, the editors and the reviewers. Any product that may be evaluated in this article, or claim that may be made by its manufacturer, is not guaranteed or endorsed by the publisher.
