# Supplementary material for: A Novel Hypothesis: A Role for Follicle Stimulating Hormone in Abdominal Aortic Aneurysm Development in Postmenopausal Women
Source: Front Endocrinol (Lausanne). 2021 Oct 13;12:726107. doi: 10.3389/fendo.2021.726107 (PMC8548664; doi:10.3389/fendo.2021.726107)
Supplement: Supplementary file 1 [file Table_1.docx]

**Supplemental Table 1.**

|  | **Study** | **Tissue/cells** | **FSHR expression** | **Detection/method** |
| --- | --- | --- | --- | --- |
| FSHR in endothelial cells | Radu *et al.* 2010 (118) | Endothelial cells of blood vessels in various tumours (prostate, breast, colon, urinary bladder, kidney, lung, liver, stomach, testis, ovary) | Protein-level | - IHC of the FSHR in endothelial cells of blood vessels in various tumours (compared with granulosa cells and Sertoli cells in normal ovary and testis tissue). Other controls for antibody specificity: immunoblotting of cell extracts from ovarian specimens and of lysates of cell cultures transfected with FSHR cDNA, and co-immunoprecipitation of radiolabelled FSH from cells that express the FSHR. |
|  | Stilley *et al.* 2014 (117) | HUVECs | Protein-level, mRNA-level | - IHC of the FSHR in endothelial cells of human umbilical cord veins (compared with granulosa cells in ovarian follicles (non-human, primate, two different animals)). - Immunofluorescence of FSHR in HUVEC (no comparison hereof mentioned with ovary tissue). - PCR of FSHR mRNA in HUVECs (compared to human ovaries). |
|  | Li *et al.* 2017 (116) | HUVECs | Protein-level, mRNA-level | - PCR of FSHR mRNA in HUVECs (compared with human granulosa cells). - Western blot of HUVECs (compared with human granulosa cells). |
| FSHR in monocytes/osteoclasts | Sun *et al.* 2006 (140) | Human and murine osteoclasts | Protein-level, mRNA-level | - Immunolabeling of mature osteoclasts (derived from bone marrow or RAW C3 cells). Ovary or testis tissue not mentioned. - PCR of FSHR mRNA in primary murine and RAW cell precursors, human osteoclasts and mesenchymal stem cells. Confirmed with Affymetrix gene arrays (FSHR in mature osteoclasts). - Western blot of human osteoclasts and mesenchymal cells (compared to COV34 ovarian granulosa tumour cells). - Immunoprecipitation of plasma-membrane and cytosolic fractions from osteoclasts to reveal a membrane location of FSHR. |
|  | Robinson *et al.* 2010 (110) | Human monocytes and osteoclasts | Protein-level, mRNA-level | - PCR of the FSHR in human monocytes and osteoclasts (compared with ovarian cells). - Western blot of the FSHR in human peripheral blood monocytes (compared with ovary tissue). |
|  | Zhu *et al.* 2012 (139) | Human monocytes and osteoclasts | mRNA-level | - PCR of FSHR in human osteoclasts and monocytes (compared to human ovarian cancer cells, COV-413). Sequence confirmation of an alternatively spliced FSH receptor transcript in osteoclasts and monocytes (compared with COV-413). |
| FSHR in hepatocytes | Song *et al.* 2016 (152) | Human and mouse liver tissue, human hepatocyte cell line HepG2 | Protein-level, mRNA-level | - IHC of the FSHR in human liver tissue (compared with human ovary tissue) and immunofluorescence analysis of FSHR in HepG2 cells. - PCR of FSHR mRNA in human/mouse liver tissue and HepG2 (compared with human ovary tissue); FSHR product was verified by electrophoresis and confirmed by sequencing (in human ovary, human/mouse hepatic tissues and HepG2). - Western blot of FSHR in human/mouse liver tissue and HepG2 cells (compared with human ovary tissue). |
|  | Guo *et al.* 2019 (153) | Human and mouse liver tissue, human HepG2 cells, mouse hepatocyte cell line NCTC 1469 | Protein-level, mRNA-level | - PCR of FSHR mRNA in human/mouse livers (compared with human and mouse ovaries), confirmed with sequencing. - In situ hybridization of FSHR in human liver tissue (compared with human ovary tissue). - Western blot of human/mouse FSHR in the liver (compared with human/mouse ovary tissue). - Immunofluorescent staining of FSHR revealed cellular localization of the FSHR in human/murine hepatocytes (ovary tissue not mentioned). - FSH-FSHR binding assay in mouse (NCTC 1469) and human HepG2 cells (compared with ovary cells, CHO). |
| FSHR in adipocytes | Cui *et al.* 2012* | Chicken adipose tissue | Protein-level, mRNA-level | - Immunocytochemical detection of the FSHR in chicken adipose tissue (chicken ovary tissue not mentioned). - PCR of FSHR mRNA (compared with chicken ovary tissue), verified by electrophoresis and confirmed by sequencing. |
|  | Liu *et al.* 2015** | Human and murine adipose tissue, 3T3-L1 mouse preadipocyte cell line | Protein-level, mRNA-level | - IHC of the FSHR on the cell membrane of human adipocytes (compared with human ovary tissue). - PCR and Western blot analysis revealed FSHR expression in human and mouse adipose tissue (compared with human granulosa cells and murine ovary tissue), confirmation by sequencing that the PCR product was the FSHR. - PCR and Western blot analysis of FSHR in 3T3-L1 mouse preadipocyte cell line (compared with mouse ovary). - Immunofluorescence of FSHR in human and mouse adipose tissue (ovary not mentioned). |
|  | Liu *et al.* 2017 (157) | Murine adipose tissue | Protein-level | - IHC of the FSHR in murine adipose tissue (no comparison with ovary or testis tissue). - Sanger sequencing confirms the expression of FSHR in adipocytes (control: sequence of FSHR in mesenchymal stem cells isolated from mouse ear lobes was identical to mouse ovarian FSHR). |
|  | Juel Mortensen *et al.* 2019† | Human-derived adipocyte cell lines (hMADS and TERT-hWA) | Protein-level, mRNA-level | - Immunocytochemistry of FSHR in both adipocyte cell lines (antibody validated in human testis tissue). - PCR of FSHR mRNA in adipocytes (compared with human testis tissue), confirmed with sequencing. |
| Stem cells | Shaikh *et al.* 2016 (97) | Murine stem cells, derived from bone marrow | Protein-level | - Immunofluorescence on bone marrow cells showed FSHR surface expression (ovary or testis not mentioned). - Immunophenotyping/flow cytometry showed that stem cells expressed the FSHR (ovary or testis not mentioned). |
| FSHR not detected | Ritter *et al.* 2008 (175) | Human male osteoclasts or RAW 264.7 cells. | FSHR not detected at protein-level or mRNA-level | - PCR did not detect FSHR mRNA in human osteoclasts and RAW 264.7 cells (compared with murine testis and ovary tissue). - Western blot of FSHR revealed no detection of FSHR in RAW 264.7 cells (compared to UMR-106 cells transfected with FSHR and murine testes and ovaries). |
|  | Allan *et al.* 2010 (174) | Murine bone, cultured osteoblasts, osteoclasts | FSHR not detected at mRNA-level | - PCR did not detect FSHR mRNA in murine bone or cultured osteoblast/osteoclast RNA preparations from spleen or macrophage-like RAW 264.7 cells (compared with murine ovary tissue). |
|  | Stelmaszewska *et al.* 2016‡ | Human umbilical cord, HUVECs or immortalized HUVECs (HUV-ST) | FSHR not detected at protein-level or mRNA-level | - PCR did not detect FSHR mRNA in umbilical cords, umbilical vein, umbilical artery, HUVEC and/or HUV-ST (compared with human granulosa tumour cells that showed FSHR exon-specific amplification bands, confirmed by sequencing; FSHR RNAscope in situ hybridization analysis confirmed negative and positive PCR results). - Immunocytochemical co-localization of FSHR in HUVECs and HUV-ST cells did not detect the FSHR (compared with human granulosa cells and HEK293 cells stably transfected with human FSHR cDNA). |

An overview of relevant studies on extragonadal FSHR expression as discussed in the manuscript. HUVEC = human umbilical cord endothelial cell. HUV-ST = SV40Tag/telomerase-immortalized human umbilical vein endothelial cell line.

Other relevant studies reporting extragonadal FSHR that have not been discussed in our manuscript are:

*Cui *et al.* FSH stimulates lipid biosynthesis in chicken adipose tissue by upregulating the expression of its receptor FSHR. J Lipid Res. 2012;53(5):909-917.

**Liu *et al.* FSH regulates fat accumulation and redistribution in aging through the Gαi/Ca(2^+^)/CREB pathway. Aging Cell. 2015;14(3):409-420.

†Juel Mortensen *et al.* Possible link between FSH and RANKL release from adipocytes in men with impaired gonadal function including Klinefelter syndrome. Bone 2019;123:103-114.

‡Stelmaszewska *et al.* Revisiting the expression and function of follicle-stimulating hormone receptor in human umbilical vein endothelial cells. Sci Rep. 2016;6:37095.
